# ggalluvial: Layered Grammar for Alluvial Plots

**DOI:** 10.21105/joss.02017

**Published:** 2020-05-21

**Authors:** Jason Cory Brunson

**Affiliations:** 1 Center for Quantitative Medicine, UConn Health

## Abstract

Alluvial diagrams use stacked bar plots and variable-width ribbons to represent multi-dimensional or repeated-measures data comprising categorical or ordinal variables (Bojanowski & Edwards, 2016; Rosvall & Bergstrom, 2010). The ggalluvial package extends the layered grammar of graphics of ggplot2 (Wickham, 2016) to generate alluvial diagrams from tidy data (Wickham, 2014).

The package makes two key contributions to the R ecosystem. First, ggalluvial anchors the imprecise notion of an alluvial diagram to the rigid grammar of graphics (Wilkinson, 2006), which lends the plots more precise meaning and opens up many combinatorial possibilities. Second, ggalluvial adopts a distinctive geological nomenclature to distinguish “alluvial plots” and their graphical elements from Sankey diagrams and parallel sets plots, which I hope prove useful as these visualization tools converge toward common standards.

## Functionality

The primary vignette thoroughly describes and illustrates the functionality of ggalluvial, and the reader is encouraged to browse the package documentation for comprehensive examples. In brief, the package contains stat and geom functions to add the following layers to a ggplot2 object:

- *strata*, or stacked bar plots, located in parallel along a (plotting) axis of (variable) *axes* or *dimensions*
- *alluvia*, ribbons through strata that connect the categories of individual cases or cohorts at different axes
- *lodes*, subdivisions of strata by their intersections with alluvia
- *flows*, segments of alluvia between strata

**Figure 1:**
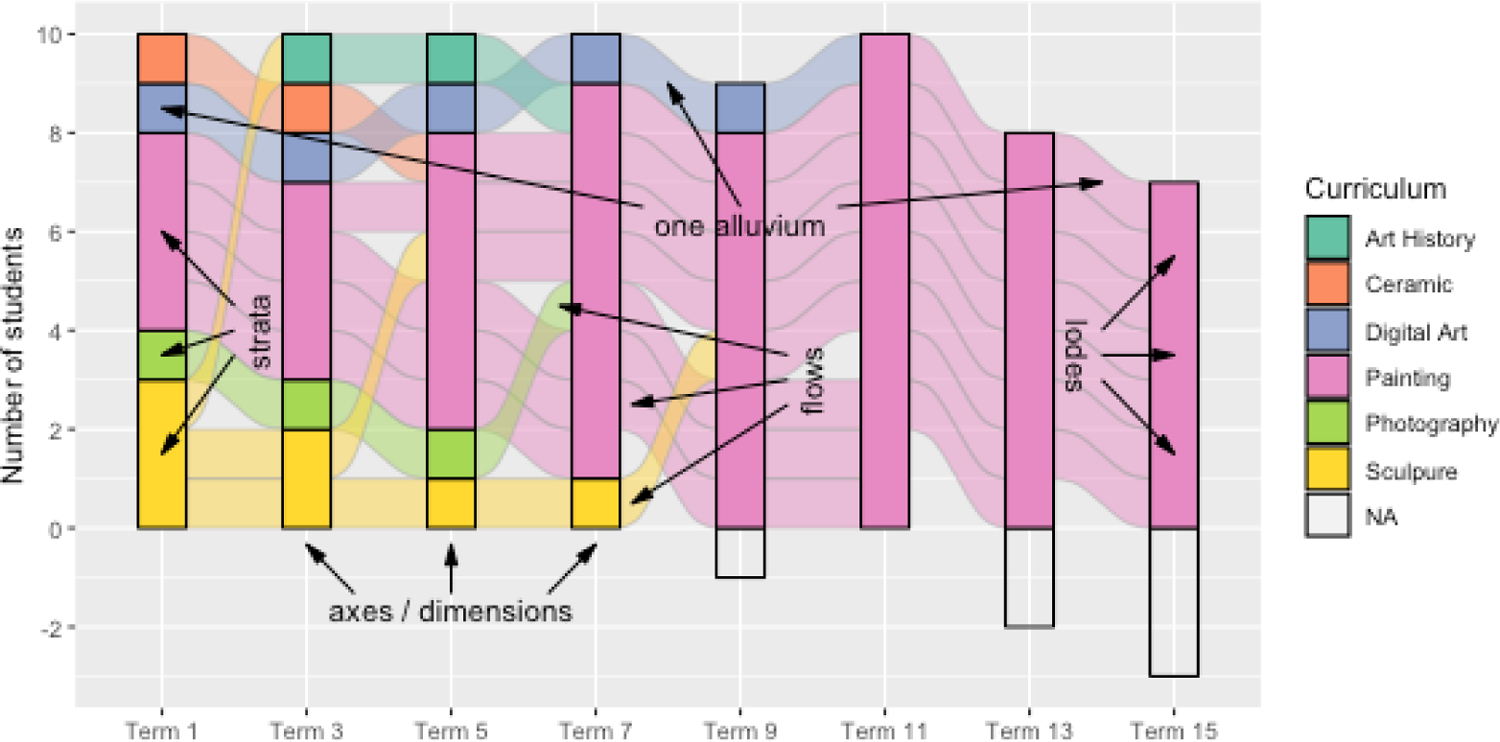
Alluvial plot of changes in curricula by a cohort of art students

Figure 1 illustrates these and other plot elements by visualizing changes in several students’ curricula (based on their declared majors) across several academic terms. Each axis corresponds to an odd-valued term (1 through 15), at which the students are grouped into strata according to their curricula—Art History, Ceramic, etc. The individual students can be tracked from term to term along their alluvia: for instance, one student started out in Digital Art, encoded by the blue ribbon, but had switched to Painting by the 11th term, where the ribbon turns pink. The partially transparent flows are colored according to their originating (not their terminating) terms, and the lodes where they intersect the strata are obscured by the solid-colored strata themselves. When a student’s curriculum is unknown, they are grouped into the “missing” (NA) stratum, which is weighted negatively in this example.

Plot layers are formed by pairing stats (statistical transformations) with geoms (mappings to graphical elements and properties); while every stat and geom has a conventional default, alternative grammatical pairings provide combinatorial richness to plotting possibilities. In the above example, the alluvium geom was paired with the flow stat, so that the flows of each alluvium could change color across the axes. Other meaningful stat–geom combinations can be found in the documentation, including pairings of the three alluvial stats (stratum, alluvium, and flow) with the text, errorbar, and pointrange geoms.

Alluvial layers can interpret tidy data in either of two formats: long (one row per lode) and wide (one row per alluvium). These are related by the pivot operations of tidyr (Wickham et al., 2019) and can be toggled between using the custom functions to_lodes_form() and to_alluvia_form(). The alluvial stats require custom aesthetics—either stratum and/or alluvium in combination with x, if the data are in long format, or some number of axis specifications (axis1, axis2, etc.), if the data are in wide format.^1^ Because the alluvial geoms are specialized to these stats, no pairings with outside stats are currently supported.

Most of the stat parameters control how the strata at each axis, and the lodes within each stratum, are ordered vertically. By default, these orderings are independent of differentiation aesthetics, so that layers are consistent within and across plots unless otherwise specified. An auxiliary vignette details the effects of these parameters. They can also be set as global options.

## Concepts

Visualizations of flow processes have long encoded magnitudes as ribbon widths, constituting a type called Sankey diagrams (Schmidt, 2008). A widely-used subtype for longitudinal categorical data represent categories as nodes threaded by edges that represent the trajectories and magnitudes of cases (Riehmann, Hanfler, & Froehlich, 2005). Their design anticipated parallel sets plots, which were adapted from parallel coordinates plots (Inselberg & Dimsdale, 1987; Wegman, 1990) to visualize multivariate categorical data, and which represent cohorts of equivalent cases as ribbons connecting categories represented as boxes (Kosara, Bendix, & Hauser, 2006). These in turn anticipated “alluvial diagrams”, proposed to visualize changes in case memberships across successive cross-sections (Rosvall & Bergstrom, 2010). Several R packages have been developed to generate diagrams of these types, including riverplot (Weiner, 2017), networkD3 (Allaire, Gandrud, Russell, & Yetman, 2017), sankey (Csárdi & Weiner, 2017), alluvial (Bojanowski & Edwards, 2016), ggparallel (Hofmann & Vendettuoli, 2013), ggforce (Pedersen, 2019), ggalluvial (Brunson, 2019), and ggpcp (Ge & Hofmann, 2019).

Sankey, parallel sets, and alluvial diagrams are often conflated, and there is currently no consensus on what features are distinctive to each type. Moreover, their graphical elements go by a variety of names, often interchangeably. In order to more clearly describe the features of ggalluvial in relation to similar packages, I have found it useful to adopt a careful demarcation among these diagram types.

*Statistical graphics* (here also simply called “plots”) are diagrams that communicate statistical information using graphical methods (Friendly, 2005) and, more narrowly, are uniquely determined from data by a fixed set of plotting rules (Wilkinson, 2006). By design, graphics produced by ggplot2 extensions are plots: The stat, geom, and other layers of a ggplot object exactly reproduce a graphic from data (under the same parameter settings).^2^ Sankey diagrams are much more flexible. The earliest engine efficiency diagrams in this tradition could take a variety of forms to depict the same energy flow and were differently annotated for different audiences (Schmidt, 2008). Software implementations may use heuristic algorithms to position their graphical elements (Allaire et al., 2017; Csárdi & Weiner, 2017) or enable users to manually, even interactively, adjust them (Allaire et al., 2017; Riehmann et al., 2005; Weiner, 2017). Paradoxically, Sankey diagrams are overwhelmingly used to represent flow, whereas the aforecited ggplot2 extensions are used to visualize a wide variety of data types. Arguably, these extensions are better understood as producing a different type of diagram.

Parallel sets plots might be viewed as a subtype of Sankey diagram with the following features: Ribbons proceed monotonically along one dimension, and every ribbon encounters a box at every axis. These graphical constraints correspond to combinatorial constraints on the data, which amount to an id–key–value structure in which every id–key pair takes exactly one value (possibly zero or missing, and optionally weighted). In this sense, the plots produced by the ggplot2 extensions (and by the alluvial package) are parallel sets plots: Cohorts are partitioned into categories at each axis and connected by ribbons whose widths encode their magnitudes.^3^

The plots produced still vary—in the shapes of ribbons, the arrangements of boxes, and the presence of gaps between boxes at the same axis. The exceptional geoms of ggparallel each offer common-angle as well as linear ribbons. Those of alluvial, ggforce, ggalluvial, and ggpcp offer one-parameter families that interpolate between straight and x-spline ribbons.^4^ The stats vertically arrange the elements (boxes and ribbons) at each axis. These distinct elements are rendered by separate layers in ggforce, ggalluvial, and ggpcp, following the additive (+) syntax of ggplot2. ggalluvial provides more levers of control over the statistical transformations, thereby over the messages conveyed by the plot, than the other packages.^5^

The ggalluvial package adopts the term *alluvial plot* for the subtype of parallel sets plots it produces, with the geological terminology introduced above.^6^ These alluvial plots are distinguished by two features: a prescribed order on the stacked elements at each axis, including both the values of the discrete variables and the ribbons connecting cases or cohorts between them; and a real-valued plotting dimension perpendicular to that of flow, along which these elements are stacked, so that gaps between them are precluded. In combination, these features confer greater meaning on the second plotting dimension.

The first feature is shared by the other packages but is not essential to parallel sets plots; such plots could, for example, arrange boxes corresponding to repeated categorical decompositions differently at different axes. While most of the packages separate boxes at each axis with gaps, these can be reduced to zero, so that each package can create alluvial plots. (ggparallel and ggalluvial alone *only* produce alluvial plots.) These features are particularly important to some applications and, in my view, can fundamentally change the way a plot is interpreted. It is for this reason that I believe the new typology and terminology are warranted.

## Applications

While most uses might be served equally well by other parallel sets plots or Sankey diagrams, alluvial plots seem exceptionally well-suited to three settings: repeated ordinal measures data, incomplete longitudinal data, and signed categorical data.^7^

### Repeated ordinal measures data.

Most Sankey, parallel sets, and alluvial implementations stack each bar plot in order of name or of size (though some follow user-provided hierarchies), and most insert gaps between categories for easy visual discrimination. Ordinal variables are most appropriately stacked in their own intrinsic and consistent order and, when the number of categories (hence of gaps) changes from axis to axis, vertical separations can obscure whether magnitude totals changed as well. A use case by Schlotter et al. (2019), to represent patients’ physical limitations following an investigational right heart valve repair technique, illustrates the use of an ordinal stratum variable (a heart failure functional classification). Another, by North, Kothe, Klas, & Ling (2019), to represent ranked preferences among several definitions of veganism by survey respondents, illustrates the importance of consistency in their order. In both cases, the fixed heights of the bar plots conveyed that no individuals were lost to follow-up.

### Incomplete longitudinal data.

Alluvial plots clearly indicate times at which longitudinal data are censored or otherwise missing: Certain strata, or the alluvia or flows connecting them, are present at one time point but absent at a previous or future one. Seekatz et al. (2018) use this feature to include in one alluvial plot a sample of *Clostridium difficile*–infected patients who had their infections ribotyped at multiple times. Patients were classified by dominant ribotype, and the alluvial plot showcased variability in this classification. While all 32 patients had at least two samples taken, only 3 had four, communicated by the shortening of the bar plots along the main dimension. Sjoding, Gong, Haas, & Iwashyna (2019) use a similar plot to trace patient groups receiving mechanical ventilation based on discretized tidal volumes, including a grey stratum for patients discontinued from intubation.

### Signed categorical data.

Edwards & Pinkerton (2019) produced a novel alluvial plot to represent changes in ownership category of owners in a halibut fishery. The total number of owners changed from year to year as exiters were not exactly matched by new entrants. In order to depict an accurate total but include both new entrants and exiters at each year, the authors affixed a negative stratum for the exiter category to each bar plot.^8^ Such a feature has no analogue in Sankey diagrams or parallel sets plots but potentially wide-ranging applications: Bar plots may use “positive” and “negative” bars to represent signed categories, such as contributors to revenue versus deficit, or to contrast the bars divided along a binary variable such as gender across age groups in a population (“pyramid plots”). Alluvial plots provide a way to track cases and cohorts across such graphics, even when cases change sign. Future applications may demonstrate additional uses for this functionality.

## Footnotes

1 Because these aesthetics are not recognized by ggplot2, they produce warnings under some conditions.

2 There are exceptions. For example, the jitter geom in ggplot2 introduces randomness to symbol positions, and the repel geoms of ggrepel (Slowikowski, 2019) use heuristic algorithms to position text.

3 The possible exceptions are the hammock plots and common angle plots of ggparallel, which are contrasted with a stricter definition of parallel sets plots than I use here, in which ribbons are straight, their widths aggregate to box widths, and they meet without overlap at the sides of boxes, partitioning them (Hofmann & Vendettuoli, 2013).

4 Several alternative curves, based on Shaffer (2019), are in development.

5 Indeed, the dependency package easyalluvial (Koneswarakantha, 2019) was built on top of ggalluvial to exchange much of this flexibility for more expedient data exploration.

6 This has the unfortunate side effect of conflating search results from the geology literature.

7 To be sure, this is a subjective assessment that may be refuted by visualization effectiveness research.

8 The authors should be credited with this innovation, which I only implemented in ggalluvial after learning about their workaround to create it using a previous version.
